# First Evidence of Placental Transfer of Ochratoxin A in Horses

**DOI:** 10.3390/toxins5010084

**Published:** 2013-01-11

**Authors:** Fiorenza Minervini, Alessandra Giannoccaro, Michele Nicassio, Giuseppe Panzarini, Giovanni Michele Lacalandra

**Affiliations:** 1 Institute of Sciences of Food Production (ISPA), National Council of Research (CNR), Via Amendola 122/O, Bari 70125, Italy; E-Mails: ale.g.vet@fastwebnet.it (A.G.); giuseppe.panzarini@ispa.cnr.it (G.P.); 2 Veterinary Clinic and Animal Productions Unit, Department of Emergency and Organ Transplantation, University of Bari, Strada Provinciale per Casamassima Km 3, Valenzano, Bari 70010, Italy; E-Mails: m.nicassio@veterinaria.uniba.it (M.N.); g.lacalandra@veterinaria.uniba.it (G.M.L.)

**Keywords:** serum, ochratoxin A, mycotoxin, placental transfer, horse

## Abstract

Ochratoxin A (OTA) is a renal mycotoxin and transplacental genotoxic carcinogen. The aim of this study was to evaluate the natural occurrence of OTA in equine blood samples and its placental transfer. For the assessment of OTA levels, serum samples were collected from 12 stallions, 7 cycling mares and 17 pregnant mares. OTA was found in 83% of serum samples (median value = 121.4 pg/mL). For the assessment of placental transfer, serum samples were collected from the 17 mares after delivery and from the umbilical cords of their foals, after foaling. Fourteen serum samples from pregnant mares contained OTA (median value = 106.5 pg/mL), but only 50% of their foals were exposed (median values = 96.6 pg/mL). HPLC analysis carried out on four serum samples (collected from two mares and their respective foals) supported the ELISA results on OTA placental transfer. This is the first report on the natural occurrence of OTA in horse serum samples and placental transfer in horses.

## 1. Introduction

Ochratoxin A (OTA) is a mycotoxin produced by several fungi of the genera *Aspergillus* and *Penicillium*, mainly by *P. verrucosum* in temperate climates and *A. ochraceus* in warm regions. In animal feed materials, the toxin is found most commonly in cereals [1]. The feedstuffs that produce the highest risks of contamination are oats and wheat and their by-products, such as bran [2,3]. Indeed, OTA is mainly concentrated in the seed coat of cereals, which is often used for animal feeding [4]. Recently, 42% of horse feed grains were contaminated by OTA at levels ranging from 0.2 to 4 μg/kg [5]. Analysis of biological fluids could provide a useful tool for assessing individual exposure to mycotoxins. Exposure to OTA may be estimated from the analysis of plasma samples because of its binding to serum albumin delays renal excretion, with consequent large interspecies differences in half-lives [1,6,7]. The horse has high economical value owing to its suitability for sport and as a domestic animal. Even though its feed composition is mainly based on cereals, it has not been considered much in studies on mycotoxin exposure and toxicity, with the exception of fumonisins. To date, no data are available on the bioavailability of OTA in the horse. Ochratoxin A is a potent renal toxin in all animal species, with pigs, dogs and poultry being particularly sensitive. Ruminants are less sensitive, due to degradation of OTA to the less toxic compound (ochratoxin α) by the rumen microflora [4]. In fact, ochratoxicosis has rarely been reported in cattle and in small ruminant species, such as sheep and goats, because microbial activity in the gastrointestinal tract can effectively and dramatically reduce OTA absorption thus protecting the animals. Herbivores, such as horses and rabbits that rely on caecal rather than ruminal fermentation, may absorb intact OTA in the small intestine and are likely to be more sensitive than ruminants [1]. 

In addition, OTA has been classified by IARC as a possible human carcinogen (class 2B). Ochratoxin A induces immunotoxicity, teratogenicity (through placental transfer), and reproductive toxicity, especially in more sensitive monogastric species, such as swine [8,9,10]. Several studies have demonstrated transplacental transfer of OTA in swine, although conflicting reports have been published: in particular, no residues in piglets of sows fed diets containing OTA at 7–16 µg/kg bw per day throughout gestation [11] or no placental transfer after ingestion of 0.38 mg/kg bw by a pregnant sow on days 21–28 [12] have been described. In contrast, Barnikol and Thalmann [13] found OTA transmission to piglets in utero when the sow was fed naturally contaminated feed and the blood concentrations in the newborn piglets were 0.075–0.12 ng/mL whereas, in the sow, it was 0.20 ng/mL. In humans, the OTA concentration in foetal serum was reported to be twice the maternal concentration, suggesting an active placental transfer of OTA [9,14,15]. To date, no data are available on OTA placental transfer in horses.

The present study focuses on the assessment of OTA occurrence and placental transfer using Enzyme-Linked Immunosorbent Assay (ELISA) on serum samples collected from 36 horses of different ages, breeds and production systems. These data were confirmed in some serum samples collected from mares and their foals by HPLC analysis. 

## 2. Results and Discussion

ELISA showed good performance and provided accurate results because the coefficients of variation obtained with standard OTA solution and serum samples were very low (2.7% and 8.3%, respectively). Despite the high cross-reactivity of the antibody toward ochratoxin C, the results with ELISA were unaffected because OTA is the major compound found in blood samples. As observed in Table 1, OTA was found in 83% of serum samples collected from horses (*n* = 36) with a mean level of 169.2 pg/mL. With respect to diet composition, the majority of horses (from 64% to 80%) fed with commercial feed, hay and oats had less OTA in serum (mean value = 150 pg/mL) than horses fed with bran (mean value = 339 pg/mL). Although different kinetic profiles of OTA in rats (and consequent bioavailability) related to gender, age, weight and fasting condition have been described [16,17,18], due to an uneven number of horses for grouping, statistical analysis was not performed on these bases. The occurrence of OTA in serum samples collected from stallions could represent a risk for equine reproductive efficiency, as it may accumulate in seminal plasma and may have a toxic effect on some functional sperm parameters, as observed *in vivo* in boar [8]. 

**Table 1:** Occurrence of ochratoxin A (OTA) levels (pg/mL) in serum equine samples assessed by ELISA.

Samples	Breeds or production system	Feed	Age (years)		Gender	OTA levels (pg/mL)
1	Pony	Hay, commercial feed	4		♂	485.1
2	Quarter horse	Hay, commercial feed	8		♂	83.5
3	Arab thoroughbred	Hay, commercial feed	8		♂	54.5
4	Saddle-horse	Bran, oats, hay	9		♂	229.9
5	Saddle-horse	Bran, oats, hay	10		♂	705.4
6	Trotter	Bran, oats, hay	12		♂	83.3
7	Quarter horse	Hay, commercial feed	13		♂	62.5
8	Saddle-horse	Bran, oats, hay	14		♂	-
9	Murgese	Hay, commercial feed	15		♂	95.8
10	Standardbred	Oats, hay	15		♂	-
11	German saddle-horse	Hay, commercial feed	17		♂	52.8
12	Trotter	Oats, hay	18		♂	347.3
13	Pony	Hay, commercial feed	2		♀	186.1
14	Pony	Hay, commercial feed	3		♀	122.9
15	Pony	Hay, commercial feed	4		♀	138.2
16	Pony	Hay, commercial feed	4		♀	-
17	Standardbred	Hay, oats	4		♀	123.6
18	Standardbred	Hay, oats	5		♀	166.7
19	Pony	Hay, commercial feed	5		♀	155.5
20	Arab thoroughbred	Hay, oats	5		♀	-
21	Quarter horse	Hay, commercial feed	6		♀	350.3
22	Standardbred	Hay, oats	6		♀	69.7
23	Saddle-horse	Hay, oats	7		♀	119.9
24	Standardbred	Hay, oats	7		♀	128.8
25	Standardbred	Hay, oats	8		♀	111.2
26	Saddle-horse	Hay, oats	8		♀	79.2
27	Standardbred	Hay, oats	8		♀	75.4
28	Standardbred	Hay, oats	9		♀	348.3
29	Quarter horse	Hay, commercial feed	9		♀	73.1
30	Quarter horse	Hay, commercial feed	10		♀	202.2
31	Quarter horse	Hay, commercial feed	10		♀	-
32	Standardbred	Hay, oats	10		♀	79.2
33	Standardbred	Hay, oats	11		♀	-
34	Standardbred	Hay, oats	12		♀	101.9
35	Pony	Hay, commercial feed	12		♀	155.2
36	Standardbred	Hay, oats	16		♀	87.9
Incidence of positive samples				30/36 (83.3%)		
Mean values of positive samples ± standard deviation				169.2 ± 145.7		
Median				121.4		
Range				52.8–705.4		

-: <50 pg/mL

Serum levels of OTA found in horses were similar to those found by other authors in swine (mean levels of 250–640 pg/mL) [19,20] but higher than those observed in chickens (mean levels of 13 pg/mL) [21]. Differences in OTA serum levels between animal species may well be related to the different half-lives of the toxin, which are strictly dependent on the affinity of its binding to serum proteins (retarding elimination and renal filtration) and to entero-hepatic circulation and biliary excretion, which might be responsible for toxin accumulation and prolonged elimination from the body [1,16,22].

Concerning placental transfer, as observed in Table 2, 83% of pregnant mares contained OTA at concentrations ranging from 69.7 to 348 pg/mL in serum samples, whereas only 50% of their foals were exposed to OTA at levels ranging from 69.5 to 252.6 pg/mL. The ratio between mare’s and foal’s OTA serum levels was variable and no correlation (*r* = −0.07) was found using Pearson’s correlation analysis. 

**Table 2:** Serum levels of ochratoxin A (pg/mL) in pregnant mares and their foals, collected from umbilical cord, assessed by ELISA.

OTA levels in pregnant mares	OTA levels in umbilical cord	Placental transfer ratio *
73.1 (29) **	-	-
202.2 (30)	-	-
128.8 (24)	-	-
111.2 (25)	-	-
119.9 (23)	-	-
69.7 (22)	-	-
166.7 (18)	252.6	1.5
123.6 (17)	-	-
- (20)	-	-
87.9 (36)	74.2	0.8
79.2 (32)	69.5	0.9
75.4 (27)	139.5	1.8
348.3 (28)	75.8	0.2
79.2 (26)	165.3	2.1
- (31)	-	-
-	-	-
101.9	96.6	0.9

-: < 50 pg/mL; * assessed as OTA levels in umbilical cord/OTA levels in pregnant mares; ** the number between brackets corresponds to the number of samples.

**Figure 1:**
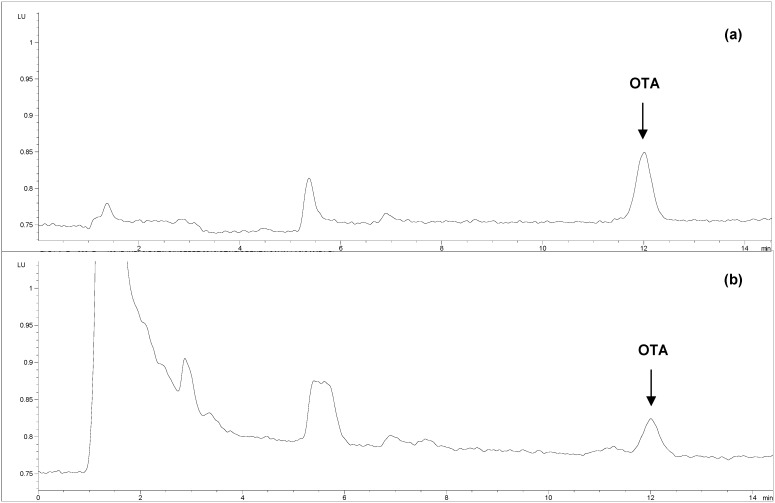
HPLC chromatograms: (**a**) standard solution of Ochratoxin A (0.025 ng); (**b**) sample of serum samples collected from mare after clean-up by immunoaffinity column (33 pg/mL).

Placental transfer of OTA was confirmed by HPLC analysis of four additional serum samples collected from mares (*n* = 2) and from the umbilical cords of their foals. The OTA occurrence was found in serum samples collected from the younger mare (7 years old) and her foal at levels of 33 and 19 pg/mL, respectively (Figure 1). The serum sample collected from the older mare (19 years old) contained OTA levels below the detection limit (15 pg/mL). The parallel presence/absence of OTA in serum samples collected from the mares and their foals might confirm the results obtained by ELISA. 

The absence of a correlation between OTA levels in umbilical and mare serum samples was in agreement with the results reported in pigs [1] after *in vivo* exposure, and could be explained by several factors, such as exposure time (gestation period and related placental vascularization) and/or placental structure. Concerning the gestation period, during the early stage of pregnancy, OTA or its metabolites could pass through the placenta into the foetal circulation and accumulate in the foetal tissues, exerting developmental toxicity, as reported by some authors [10,23]. A reduced blood supply to the placenta or foetal tissues during the latter stages of foetal development might prevent or interfere with the access of toxin to umbilical blood [23]. Concerning placental structure, swine and equine placenta are characterized by a chorionic epithelium which could prevent or interfere with the access of toxin to the umbilical blood *in vivo* and consequent OTA foetal uptake, strictly related also to the developmental stage of the placenta [1,22]. Concerning other animal species, the few data published on OTA placental transfer in ruminants have shown very low penetration by OTA through the placenta; in fact, after intravenous administration of a high dose of OTA (1 mg/kg body weight) to pregnant ewes, OTA was not detected in the amniotic fluid and foetal tissues and levels were 400- to 1000-fold lower than in the maternal blood [24].This animal species has a syndesmochorial placenta, *i.e.*, the epithelium of the chorion is in contact with the connective tissue of the lamina propria of the uterine mucosa, lacking the epithelium on the maternal side. By contrast in humans, the haemochorial placenta allows direct contact between the maternal blood and the foetal chorion and foetal blood is separated from the maternal blood circulation by polarised cells, which possess carrier-mediated transport pathways, similarly renal proximal tubules and intestinal epithelial cells [9]. For this reason, Miraglia *et al.* [14] showed higher OTA levels in placenta and in funiculum than those found in the corresponding maternal serum and a slight correlation was reported between umbilical and maternal serum OTA levels [25]. Postupolski *et al.* [15] found a statistically significant difference (*p* < 0.05) between the mean OTA concentration in maternal blood serum (1.14 ng/mL) and umbilical cord serum (1.96 ng/mL). The mean ratio calculated for OTA maternal blood/foetal blood was 1.96 ± 0.94. As suggested by Postupolski [15], the higher OTA content in foetal blood could be explained as being due to its active transport across the placenta, perhaps as a consequence of its chemical characteristics (such as the similarity between the chemical structures of OTA and phenylalanine, low molar mass, lipophilic character, serum binding property). Consequently, particularly during the first five months of life, the foetus was more sensitive to xenobiotics (such as OTA) mainly due to insufficiently adequate function of the kidneys and hepatic metabolism systems [15]. 

## 3. Experimental Section

### 3.1. Collection of Serum Samples

Serum samples were collected from a population made up of 12 stallions, 7 cycling mares and 17 pregnant mares from July to October 2008. The pregnant mares were housed at the “Pegasus” Equine Reproduction Centre in Bari (Italy) for a variable period (from 2 to 20 days) until delivery. After delivery, blood samples (almost 10 mL) were collected from foaling mares (17) and, at the same time from the umbilical cord or jugular vein of the corresponding foal (*n* = 17). The collection of blood samples from the other animals was carried out on farms located in the Apulia region. Serum samples were obtained by centrifugation (speed: 2500 rpm, gravity: 664 *g*) of blood samples using an Eppendorf 5804 R centrifuge (Eppendorf, Hamburg, Germany).

History concerning information on age, gender, feed and breeds or production systems was gathered and is shown in Table 1. 

### 3.2. Determination of OTA in Serum Samples by ELISA and HPLC

The Ridascreen ochratoxin A 30/15 test (R-Biopharm; Milan, Italy) is a competitive enzyme immunoassay for the quantitative analysis of OTA in serum samples, with a detection limit of 50 pg/mL. R-Biopharms recently performed an in-house validation for the analysis of bovine and pig serum samples, giving recovery values of 110%–114%. The cross-reactive percentages were 100%, 44%, 14% and <0.1% for OTA, Ochratoxin C, B and α, respectively. Samples were extracted according to the ELISA kit manufacturer’s instructions. The operating range of the standard curve was from 50 to 1800 pg/mL. OTA concentrations in serum samples were assessed using specific RIDA SOFT Win software provided by the company. In addition, four serum samples collected from mares (7 and 19 years old, respectively) and their foals were analyzed by HPLC with a fluorescence detector. Samples were extracted by following the Ridascreen protocol and the extracts purified through OchraTest immunoaffinity columns (Vicam, A Waters Business-Milford, MA, USA). This involved diluting 2 mL of serum sample with 2.5 mL of 1 N HCl and 4 mL of dichlormethane (in a centrifugal screw cap vial). The mixture was shaken for 5 min and centrifuged for 15 min at 2360 *g* at 15 °C. Two mL of dichloromethane layer was transferred into another centrifugal screw-cap vial and extracted with 2 mL of 0.13 M sodium hydrogen carbonate buffer by shaking for 5 min and centrifuging for 15 min at 2360 *g* at 15 °C. The upper sodium hydrogen carbonate layer was collected in a clear screw-cap vial and the extraction and centrifugation steps were repeated on the dichloromethane layer. Both sodium hydrogen carbonate layers were combined and diluted with 0.75 mL of 1 N HCl and 2 mL of dichloromethane. The mixture was mixed for 10 min by shaker and centrifuged for 5 min at 2360 *g* at 15 °C. The dichloromethane layer was recovered and evaporated to complete dryness at 60 °C. The dry residue was redissolved in 1 mL of 0.13 M hydrogen carbonate buffer. One mL of residue (equivalent to 1 mL serum) was cleaned up through an OchraTest immunoaffinity column (Vicam, Franklin, TN, USA) at a flow-rate of about one drop per second. The column was washed with 2 mL solution containing NaCl (2.5%) and NaHCO_3_ (0.5%) followed by 2 mL distilled water at a flow-rate of 1–2 drops per second. OTA was eluted with 2 mL methanol and collected in a silanized vial. The eluted extract was evaporated under a nitrogen stream at *ca*. 50 °C and reconstituted with 250 μL of a solution of acetonitrile-water-acetic acid (99 + 99 + 2, *v*/*v*/*v*).

One hundred microliter volume of reconstituted extract (equivalent to 0.4 mL serum) were injected into the chromatographic system (Agilent 1100 Series, Agilent Technologies, Santaclara, CA, USA ) The following chromatographic conditions were used: fluorescence detector set at excitation wavelength 333 nm and emission wavelength 460 nm; HPLC column Symmetry^®^ C_18_ (150 × 4.6 mm, 5 μm particle size; Waters, Milford, MA, USA) preceded by a 0.5 μm Rheodyne guard filter; mobile phase of acetonitrile-water-acetic acid (45 + 55 + 1, *v*/*v*/*v*); flow rate, 1 mL/min. The LOD of the method was 15 pg/mL (*S*/*N* 3:1).

## 4. Conclusions

This study for the first time reports data on the natural occurrence of OTA in serum samples and placental transfer in horses. Levels of OTA found in serum samples could represent an additional risk for this monogastric animal potentially exposed to mycotoxins through cereal feeding. 
